# Impact of toll-like receptors on food allergy: mechanisms involved and scientific evidence

**DOI:** 10.1007/s13105-025-01111-9

**Published:** 2025-09-03

**Authors:** Sergio Álvarez-Azcarreta, Wissal Mountasar-Didouch, Adrián Macho González, Francisco José Sánchez Muniz

**Affiliations:** 1 Student of the subject “Nutrition: Diet-Gene Interaction”, Degree in Human Nutrition and Dietetics, Complutense University of Madrid, Madrid, Spain; 2 Department of Nutrition and Food Science, Faculty of Pharmacy, Complutense University of Madrid, Ramón y Cajal Square S/N. 28040, Madrid, Spain; 3 AFUSAN Research Group, Sanitary Research Institute of the San Carlos Clinical Hospital (IdISSC), Madrid, Spain

**Keywords:** Food allergies, Diet, GALT, Microbiota, TLRs, Genes

## Abstract

Food allergy (FA) is an exacerbated immune system response to harmless food antigens following sensitization. The incidence of FA has risen significantly over the past two decades, a trend often attributed to modern lifestyle factors such as dietary patterns, antibiotic use, and urban environments. Sensitization may result from a compromised intestinal barrier caused by inflammatory bowel diseases, genetic predisposition, or a combination of both. These conditions trigger an inflammatory response involving mechanisms such as the activation of Toll-Like Receptors (TLRs), which recognize pathogen-associated molecular patterns. This review examines the intestine's role as a key antigen-sensing organ through three critical components: a) gut-associated lymphoid tissue, b) the mucosal immune system, and, c) the intestinal microbiota in the development of FA. The role of TLRs (particularly TLR2 and TLR4) in recognizing bacterial membrane-derived compounds (e.g., lipopolysaccharides) and how commensal bacteria generate TLR ligands that influence allergen sensitization *vs*. tolerance is discussed. The importance of candidate gene polymorphisms encoding TLR proteins and other molecules associated with tolerance and sensitization to food antigens is also commented on. Finally, future research directions and preventive strategies to mitigate FA risk and development are suggested.

## Introduction

Food allergy (FA) is a significant public health problem, with its prevalence having increased markedly over the last 10–20 years. Current estimates suggest that FA affects approximately 8% of children and 10% in adults [4]. However, obtaining precise epidemiological data remains challenging, particularly in developing countries and rural areas, where limited access to healthcare and diagnostic resources impedes data accuracy [64]. Several factors are involved in the FA increase prevalence, primarily related to lifestyle modifications. This included changes in the quantity and quality of dietary intake, increased urbanization and associated hygiene practices, widespread use of antibiotics and other medications, and vaccination programs that may reduce the activation of innate immunity. Collectively, these factors can contribute to increased sensitization and the development of FA [4, 24, 35].

FA negatively impairs patients’ life quality, as they require lifelong control of food consumption to avoid oral contact with allergenic compounds [4, 71]. While any food can cause an allergenic reaction, only a limited number of them are responsible for inducing or increasing susceptibility to FA (Table 1). Among plant-based proteins, lipid transfer proteins are the main allergens associated with FA in Mediterranean populations, though their prevalence is rising in other European countries as well [64, 71]. Foods containing these include peanuts, some cereals like wheat, and nuts, although it is important to highlight that foods from animal origin such as milk, eggs, fish, and shellfish also contain proteins in their composition that can act as allergens in a significant percentage of individuals and cause FA [49, 50, 64].

**Table 1:** Main food allergies, food sources, and potential effects on nutrient deficiency

Food allergies	Main allergens	Food sources	Nutrients and bioactive compounds affected
Cow's milk proteins	Caseins, α-lactalbumin, β-lactoglobulin	Butter and margarine of animal origin, milk, cream, yogurt, ice cream, dairy desserts and processed foods containing milk or butter	Proteins, vitamins A and D, riboflavin, pantothenic acid, cyanocobalamin, calcium, magnesium, phosphate
Soy Legumes (chickpeas, lentils, others)	Gly m1-Gly m6 Len c1, Len c2, SBP55	Miso, edamame, natto, tofu, tempeh, okara, and soy derivatives (cheese, milk, flour, lecithin, protein isolate, sauces, etc.) Azuki, fava, chickpeas, green peas, beans, lentils, lupins, mung beans, navy beans, and peanuts	Plant protein, folates, iron, zinc, calcium, dietary fiber, isoflavones
Rice	Orys s1	Rice and derived products (flour, milk, oil, soups, etc.), sake, manufactured products containing rice (cookies, snacks)	Carbohydrates, proteins, vitamins B1, B2, B3, iron, zinc, manganese, copper, ferulic acid, α-tocopherols, tocotrienol, linoleic acid
Eggs	Ovomucoid and conalbumin (white) and albumin (yolk)	Egg white and yolk, cakes, biscuits, special breads, mayonnaise	Proteins, vitamins A, D, E, B2, B12; pantothenic acid, biotin, selenium, iodine, phosphate
Potatoes and vegetables	Patatin	Potato, mashed potatoes, processed potato products, tomatoes, lettuce, canned vegetables, and frozen vegetables for stews	Carbohydrates, vitamins B6, C, folates, potassium, phosphorus, calcium, magnesium, manganese, iron, zinc, copper, flavonoids, anthocyanins, carotenoids
Fruits	Citrin	Citrus, apple, fruit juices and lemon seeds. Cross allergy with cashew	Carbohydrates, fiber, vitamins B1, B6, B9, C, E, A, potassium, calcium, phosphorus, magnesium, sodium, flavonoids, carotenoids, limonene, imonoids, synephrine
Corn	LTP	Flour, corn products (flour, oil, starch, corn syrup), baked goods with corn, dextrins, maltodextrins, dextrose	Vitamins B1, B2, B3, B5, C, A, K, selenium, β-carotenes, phytosterols, and polyphenols (in roasted corn)
Cereals (wheat, rye, barley, oats, spelt, triticale)	Gluten, gliadin, glutenin, avidin, hordenine	Bakery products, pasta, cakes, and Mexican food	Proteins, carbohydrates, thiamine, riboflavin, niacin, iron, chromium
Meat and meat products	Gal d7 (chicken), albumins and immunoglobulins	Chicken meat, meat products, products based on or containing meat (pizzas, rice dishes, pies)	Protein, iron, zinc, vitamins B12, vitamin B3, pantothenic acid, vitamin B6, coenzyme Q10, taurine, conjugated linoleic acid, glutathione, lipoic acid, betaine, L-carnitine, creatine, carnosine and anserine
Fish	Parvalbumin	All types of fish and derivatives (sauces, soup, gelatin, caviar, surimi, etc.)	Proteins, iodine, calcium, phosphorus, selenium, fluoride, Vitamins, A, D, omega-3 fatty acids; EPA and DHA
Seafood	Tropomyosin	Crab, lobster, shrimp, prawns, mollusks, soups, rice dishes, and pizzas	Proteins, iodine, copper, iron, zinc, fluoride, selenium
Nuts (including peanut)	Ara h1, Ara h2, Ara h3	Peanut (oil, flour and butter), confectionery, frozen desserts, Asian dishes made with nuts	Vitamin E, niacin, magnesium, linolenic acid (walnut), others (depending on the nut)

*DHA,* docosahexaenoic acid; *EPA,* eicosapentaenoic acid; *LTP,* lipid transfer protein. Table made from [38, 42]

Though remarkable progress has been made in FA treatment, particularly with allergen neutralization and vaccine use, there is currently no specific curative therapy available, largely due to the incomplete understanding of the genetic basis and immune mechanisms involved in this disease [3, 8]. Standard therapy remains the elimination of the causative dietary allergen. However, diagnostic confirmation of FA is required, including a detailed clinical history, physical examination, and laboratory tests-most notably, the oral food challenge, which involves the controlled introduction of the suspected allergen [48], in order to avoid unnecessary elimination of foods. The most innovative therapy to date is food desensitization or oral immunotherapy (OIT) which involves the gradual introduction of allergenic foods to induce tolerance [3, 52], that will be discussed further in this review.

The complexity of FA, primarily involves, from a preventive perspective, demands detailed knowledge of foods and food products, mostly of industrial origin. Therefore, it is essential to educate and provide nutritional training to patients and their families enabling them to understand food labels, assess restaurant meals, and identify risk behaviors that can lead to unexpected allergenic reactions. Furthermore, patients at risk of anaphylaxis should receive specific training to promptly recognize early symptoms and respond appropriately [46, 50]. Management of FA also necessitates the withdrawal of allergenic foods many that are commonly considered dietary staples. This restriction can pose health risks, potentially leading to marginal or clinical nutritional deficiencies. To mitigate these risks, it is important to identify alternative food sources that provide the necessary nutrients.

Table 1 outlines the nutrients and bioactive compounds that may be affected when allergenic foods are removed from the diet.

## Immune system of the intestinal mucosa

The intestine is one of the most extensive organs in vertebrates, whose main function is food digestion to obtain nutrients and bioactive compounds through chemical mechanisms (such as enzymes, pancreatic juice, and bile) and mechanical processes (including segmentation, haustral contraction, and mass movements). These processes are controlled and regulated by neurotransmitters, hormones, and endocrine, exocrine, and paracrine secretions; some of which originate from specialized cells within the gastrointestinal tract itself [42].

**Fig. 1:**
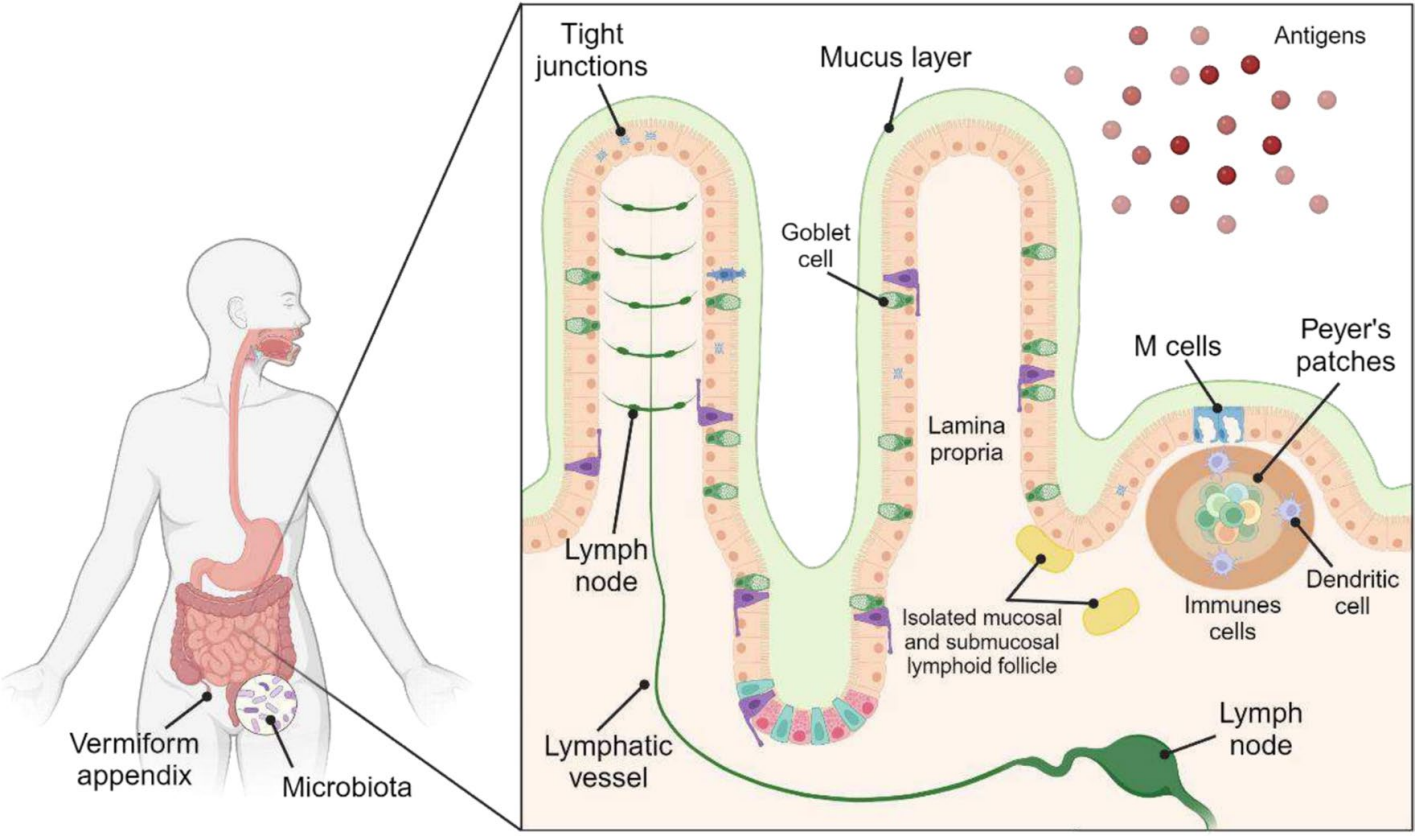
Immune system of the intestinal mucosa. Notice the presence of vermiform appendix, Peyer’s patches, lymphoid follicles, nodes, and different cell types. Created in BioRender

The intestine is continuously exposed to various infectious, allergenic, and carcinogenic agents [45]. Consequently, the gastrointestinal tract contains the largest quantity and diversity of immune compartments and immune cells in the body. These characteristics enable it to control the potential entry of pathogens and foreign substances, such as food antigens, and to determine whether or not to induce an immune response. These processes are mainly carried out in the gut-associated lymphoid tissue (GALT), a key site for antigen sampling and adaptive immune induction within the intestinal wall [42, 45, 52]. GALT includes Peyer's patches (PP), primarily in the ileum, the vermiform appendix, and the numerous isolated lymphoid follicles distributed throughout the intestine (Fig. 1), whose most important aspects are detailed below.

### Peyer's patches

PP are regions dedicated to adaptive immune preparation, specialized in the efficient initiation and propagation of immune responses. They are located in the walls of the small intestine, with a higher density in the ileal area, where a lymphoid ring can be found at the ileocecal junction (Fig. 1). In experimental mice, it has been observed that the luminal surface of PP is covered by a follicle-associated epithelium, which is coated with mucus and rich in specialized epithelial cells known as M cells. The primary function of M cells is to “capture” antigens, such as bacteria, viruses, and secretory immunoglobulin A (IgA) bound to antigens. This is accomplished through transcytosis into the parenchyma of PP. M cells form “pockets” that facilitate direct interaction with immune cells located in the subepithelial dome. The subepithelial dome is dominated by dendritic cells (DC) and other myeloid cells, and also contains memory CD4 + T lymphocytes as well as B lymphocytes expressing IgA, IgM, and IgG isotypes. Additionally, B cells in this area secrete antibodies in response to cytokine stimulation. PP are associated with B cell maturation, supporting both clonal expansion and somatic diversification of the marginal B cell system [42, 45].

### Vermiform appendix

The human vermiform appendix has long been considered a vestigial organ; however, it is currently proposed to serve as a “sanctuary” or “reservoir” for intestinal bacteria, allowing for the restoration of gut microbiota following episodes of diarrhea or changes in intestinal peristalsis. Within the appendix, naive T lymphocytes, CD4 + T cells, and B cells at different stages of development can be observed, as well as memory B lymphocytes located in the subepithelial dome. These observations suggest that this vestigial organ has an important, if not essential, role in the adaptive immune system [42, 45].

### Isolated lymphoid follicles

Isolated lymphoid follicles are circumscribed, compact aggregates of lymphocytes that form discrete lymphoid structure with a diameter ranging from 0.1 and 1.3 mm. They are significantly smaller than PP or the follicular chain of the vermiform appendix and are distributed throughout the intestinal tract. Their cellular composition, density, and location vary depending on the intestinal segment. Based on their anatomical location, two distinct types can be differentiated: mucosal and submucosal isolated lymphoid follicles (Fig. 1). These follicles are primarily composed of B lymphocytes, which are surrounded by inducer cells, DC, and stromal cells. These B lymphocytes ultimately differentiate into IgA-producing plasma cells [42, 45].

## Accept or attack? The body’s response to dietary antigens

Diet is the primary source of antigens/allergens in FA. The intestinal barrier plays a pivotal role in controlling luminal antigens translocation, guiding the GALT and maintaining the delicate balance between immune tolerance and sensitization [71]. Since intestinal epithelial cells are the primary defense barrier against antigens, their integrity is essential to shield the mucosal immune system from overexposure to dietary protein antigens, bacterial contamination, or other antigenic substances. This barrier function relies on tight junctions (Fig. 1), multiprotein complexes that form a selectively permeable seal between adjacent epithelial cells. These junctions regulate the paracellular passage of metabolites, water, and ions, through specialized proteins such as claudins, occludins, and zonulins [64, 71]. However, disruption of paracellular transport can allow food antigens to cross the epithelial barrier, initiating allergic sensitization. For instance, gliadin -a wheat protein- can disrupt occludin-zonulin interactions, increasing intestinal permeability. This enable food antigens to enter the bloodstream, potentially triggering the sensitization process that culminate in rejection [50, 51].

To ensure a healthy state, the immune system must differentiate between pathogenic environmental antigens and harmless environmental antigens. This requires the induction of immune tolerance mechanisms, a state of unresponsiveness to common food antigens. In subjects affected by FA, sensitization to food allergens produces inappropriate inflammatory immune responses to allergenic foods, resulting in the loss of immune tolerance and the onset of sensitization or immunointolerance [71]. Conversely, desensitization to food allergens involves therapies that enable intolerant individuals to temporarily increase their reactivity threshold to allergens. Through appropriate and gradual exposure, the immune system adapts, reducing the likelihood of allergic reactions. However, this process often fails to achieve a sustained, long-lasting response, as the immune system may revert to its sensitized state without continued intervention [71].

### Tolerance

Although, it is widely accepted that most antigen recognition processes occur in the intestines; approximately 2% of dietary proteins bypass the intestinal epithelium intact. These proteins can be transported directly or indirectly to the liver and/or secondary lymphoid tissues where they undergo antigen presentation by specialized immune cells [11]. As previously described, immune tolerance is characterized by the absence of a sustained immune response to potential food allergens, even without regular exposure. This tolerance status originates at the GALT in the small intestine [50], where dietary antigens are first encountered and processed.

**Fig. 2:**
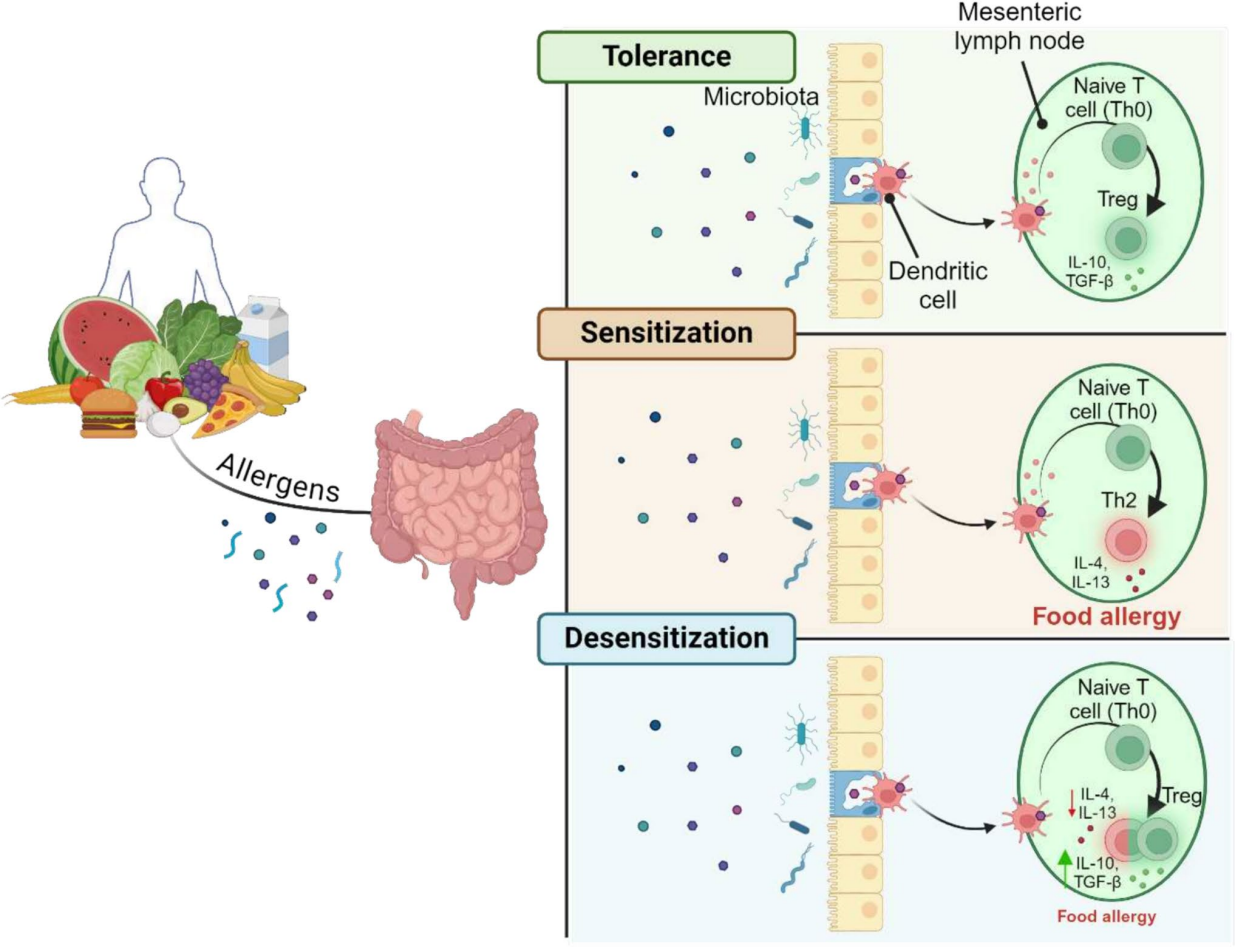
Tolerance, sensitization, and desensitization to food antigens. Representative figure of the mechanisms involved in the processes of tolerance, sensitization, and desensitization to food antigens. Once the antigen reaches the lamina propria layer in the intestine, DC transport food antigens to the mesenteric lymph nodes where tolerance can occur (naive T cells promote their differentiation into Treg cells, capable of producing immunoregulatory cytokines such as TGF-β and IL-10), sensitization (although the mechanism involved is unknown, naive T cells promote their differentiation into Th2 cells, producing a large amount of proinflammatory cytokines such as IL-4 or IL-13) or desensitization (continuous exposure to allergen doses leads to anergy and/or elimination of Th2 and an increase in Treg cells, including CD4 + T cells producing IL-10 and TGF-β) can occur. TGF-β, transforming growth factor β. Created in BioRender

To maintain immune tolerance, numerous cell types are involved in a) the antigen pass from the intestinal lumen to the lamina propria and lymphoid tissue, b) the antigen presentation and induction of T cell responses in lymphoid tissue, and c) the subsequent return of immune effector cells to the intestine (Fig. 2). Food antigens present in the intestinal lumen cross the intestinal epithelium by paracytosis or transcytosis [69] and are captured further by specialized cells, such as M cells lining the GALT or by DC and/or macrophages [71]. Cell-mediated trafficking of antigen to secondary lymphoid tissue promotes the tolerance establishment, although the relative importance of mesenteric lymph nodes, PP, and different types of secondary lymphoid tissue has not been fully established yet.

Once the antigen reaches the intestinal lamina propria, the CD103 + DC transport food antigens to the mesenteric lymph nodes, where they interact with naive T cells and promote their differentiation into regulatory T cells (Tregs). After their generation in the mesenteric lymph nodes, Treg cells upregulate CCR9 and integrin α4β7 expression to return to the lamina propria, where they expand locally to induce tolerance. The presence of large numbers of Treg cells in the intestine depends on the presence of food antigens. Therefore, these cells are crucial for producing non-inflammatory IgA responses, inducing anergy in effector T cells and inhibiting proinflammatory cells such as eosinophils, basophils, and mast cells, resulting in oral tolerance. Specifically, within the intestine, various Treg subtypes—both intestinal and peripheral Treg—play a crucial role due to their capacity to produce immunoregulatory cytokines such as transforming growth factor β (TGF-β) and IL-10, both of which are essential for Treg cell functions [71]. Additionally, the gut microbiome and the dose and timing of antigen exposure, are also likely to influence the development of oral tolerance [3].

### Sensitization

DC play a dual role in immune regulation: they orchestrate both tolerance/protective responses and sensitization pathways that sustain allergic disease. This occurs primarily through excessive Th2 polarization and/or impaired Treg generation, which disrupt immune homeostasis.[42, 45].

While the precise etiology of FA remains unclear, aberrant innate immune activation drives Th2 differentiation upon dietary allergen exposure. This process is marked by: Reduced Treg populations, expansion of allergen-specific Th2 cells with IgE class switching, and activation of downstream allergic effector pathways [3]. Furthermore, it has been proposed that microbial exposure (inducing both Th1 and Th2 polarization) typically promotes Treg-mediated immune regulation. However, reduced microbial contact in modern environments diminishes Treg production, impairing control over immune responses to harmless antigens like food allergens [69, 71]. Importantly, allergic disorders cannot be attributed solely to Treg dysfunction but rather involve multifactorial immune dysregulation [69, 71].

### Desensitization

 OIT involves the controlled administration of gradually increasing allergen doses to reduce reactivity to natural exposure [71]. Although some studies clearly suggest the benefits of short-term desensitization, permanent desensitization remains controversial due to inconsistent clinical outcomes. Current guidelines emphasize cautious allergen avoidance even after therapy [3, 11, 71]. As summarized in Fig. 2, a breakdown of natural oral tolerance shifts the balance from Treg-mediated suppression to allergen-specific Th2 dominance, driving FA pathogenesis. During OIT, continued exposure to allergen doses leads to Th2 anergy and/or elimination and increase of Treg cells, with expansion of IL-10-producing CD4 + T cells. These changes promote food allergen acceptance and suppress allergic responses through immunomodulatory mechanisms [3].

## Types of food allergies

FA is defined as an immune reaction to food proteins that may or may not be mediated by IgE. From an etiological perspective, FA are very different from food intolerances [64]. According to the European Academy of Allergy and Clinical Immunology (EAACI), FA causes non-toxic adverse reactions that vary among individuals and typically result from immune mechanisms. In contrast, food intolerances are considered toxic reactions that depend on the specific food involved and do not show evidence of immune system involvement [46]. FA are classified as non-toxic adverse reactions triggered by an immune response following repeated exposure to a particular food [71].

Immunocompetence, or the ability to react against foreign substances, is subdivided into innate immunity, which provides immediate defense against antigens, and acquired immunity, which is specific and develops with exposure to various antigens. Food antigens are generally proteins with a molecular weight between 8 and 80 kDa and are responsible for eliciting allergic reactions [18]. FA are hypersensitivity reactions linked to “inadequate” immunological mechanisms in the presence of one or more allergens that reach the immune system through different routes: inhalation (respiratory), ingestion (digestive), or skin contact. Hypersensitivity reactions are further classified based on the immunoglobulin involved, and four types are described. Type I is mediated by IgE, while types II and III are normally mediated by IgG, and type IV reactions do not involve IgE [18].

### Type I

These are characterized by the development of IgE against food allergens (e.g. milk, egg, peanut). Upon initial contact with the allergen, IgE binds to the Fragment crystallizable (Fc) region of receptors on the membranes of tissue mast cells and circulating basophils, sensitizing these cells to the allergen. Subsequent exposure causes these sensitized cells to undergo degranulation, releasing pharmacological mediators such as histamine, leukotrienes, and prostaglandins, normally causing vasodilation, smooth muscle contraction and sometimes inflammation. In this reaction, the intervention of mediators such as IL-3, which stimulates mast cell proliferation, IL-4, and IL-13, which stimulate and increase the response of Th2 cells, and IL-14, which stimulates and increases the response of Th2 cells, have been proposed. IL-3, IL-5, and GM-CSF (granulocyte–macrophage colony-stimulating factor) that stimulate the production and activation of eosinophils; tumor necrosis factor α (TNFα), promotes inflammation, cytokine production, and cellular proliferation; and macrophage inflammatory protein 1α (MIP-1α) induces chemotaxis [62].

### Type II and III

They are involved in forming immune complexes between cell surface antigens and IgG or IgM antibodies. These complexes activate the classical complement pathway, initiating a cascade that leads to the elimination of the cells presenting these antigen–antibody complexes [62].

### Type IV

This hypersensitivity reaction mainly affects T cells and is associated with some disorders, such as celiac disease in genetically predisposed individuals [59]**.** Celiac patients often exhibit high concentrations of antibodies not only against gluten, but also anti-gliadin, anti-reticulin, anti-endomysium, and/or anti-transglutaminase. The symptoms resulting from an allergic reaction to a particular food can vary widely. Importantly, these symptoms depend less on the amount of food ingested and more on the specificity of the antigen, the individual’s genetic predisposition, and other factors such as age, infections, stress, menstrual cycle, and the site of antigen exposure. [51, 62].

## TLRs: structure, function, and immunological relevance

Toll-like receptors (TLRs) are type I transmembrane receptors play a critical role in the initiation of the innate immune response, primarily through pathogen recognition [15]. The discovery of TLRs has revolutionized immunology by bridging the gap between the innate immune pathogen detection and the adaptive immune response. This connection occurs through the regulation of antigen-presenting cells (APCs) and key cytokines. Recent studies suggest that TLR signaling pathways directly regulate the activation, differentiation, development, and function of T cells across different physiological conditions [6, 67].

TLRs are expressed in different immune and non-immune cells such as B cells, natural killer (NK) cells, DC, macrophages, fibroblasts, endothelial cells, but also in mast cells, mononuclear phagocytes, and T lymphocytes [18, 61]. TLRs detect pathogen-associated molecular patterns (PAMPs) and other stimuli, initiating signaling cascades that drive immune cell activation and cytokine production. These processes are essential for innate immunity, providing first-line defense against bacteria, viruses, and other microorganisms, even in the possible absence of preexisting immunological memory.[15, 18].

In humans, ten functional TLRs (TLR1–10) have been identified, each selectively recognizing different PAMPs [18, 67]. TLRs are classified by their subcellular localization in: extracellular-TLRs (TLR1-2, TLR4-6 and TLR10) or intracellular-TLRs (TLR3, TLR7-9), primarily located within endosomal compartments (Table 2). However, recent studies, highlight that intracellular TLRs are also expressed on cell surfaces [67]. Structurally, these receptors are composed of a horseshoe-shaped region, which performs the recognition with the target PAMP, a transmembrane domain and another cytoplasmic TOLL/IL-1 receptor (TIR) domain, responsible for initiating the signaling cascade, recruiting proteins with the TIR domain, which normally leads to the activation of transcription factors such as NF-κB and AP-1 activating protein and trigger the inflammatory response. Most TLRs are homodimers, while TLR2 and TLR10 form heterodimers with each other or with TLR1, TLR2 and TLR6. The functional relevance of heterodimerization involving TLR10 remains to be fully defined [7].

**Table 2:** Expression of TLRs in different immune cells and their main pathogen-derived activators

TLR	PAMP	Immune cells
TLR1/TLR2	Triacyl lipopeptides	Most cells, including DC
TLR2	Lipoproteins, peptidoglycans, zimosans, lipoarabinomananns, porins	Peripheral mononuclear leukocytes, DC, monocytes, and T cells
TLR3	Double-stranded RNA virus	DC, NK, and T cells
TLR4	Lipopolysaccharides, HSP70	Macrophages, DC, and T cells
TLR5	Flagellin	Monocytes, DC, NK, and T cells
TLR6/TLR2	Diacyl lipopeptides	High expression in B cells and DC; low in monocytes and NK cells
TLR7	Single-stranded RNA virus	B cells, DC, NK, and T cells
TLR8	Single-stranded RNA virus	Monocytes, DC, NK, and T cells
TLR9	DNA CpG	DC, B cells, PBMC, macrophages, NK, and microglia cells
TLR10	Unknown	B cells, DC, monocytes, and T cells

*DC,* dendritic cells; *DNA CpG,* unmethylated CpG sequences in DNA; *HSP70*, 70 kilodalton heat shock protein; *NK*, natural killer cells; *PAMP*, pathogen-associated molecular patterns; *PBMC*, peripheral mononuclear leukocytes; *TLR*, toll-like receptor. (Table modified from [17])

TLR expression profiles vary widely depending on the cell type, tissue region, developmental stage, or health status. For instance, the intestinal epithelium demonstrates an uneven distribution of these receptors between the small intestine and the colon [67]. Huhta et al. [23] observed increased expression of TLR3-TLR5, TLR7, and TLR8 in the human small intestine compared to the large intestine, whereas Price et al. [57] reported the opposite pattern in a mouse model.

### Mechanism of action of TLR

**Fig. 3:**
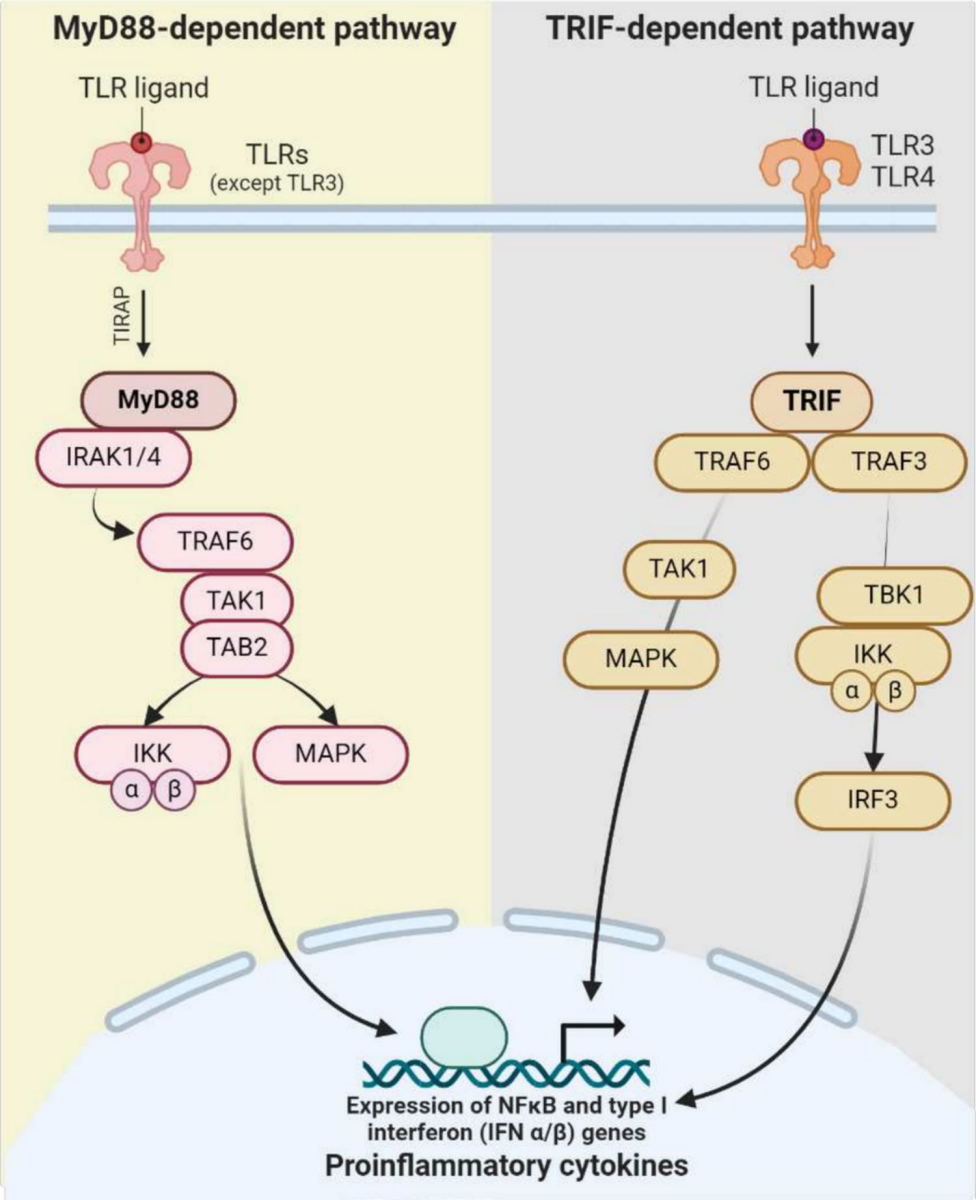
TLR signaling pathways. Activation of TLRs through the MyD88-dependent pathway induces the formation of the MyD88 complex, which contains IRAK1 and IRAK4. This activation of IRAK1 induces the activation of TRAF6 and TAK1/2, which promotes the activation of the IKK and MAPK complex, inducing the expression of proinflammatory genes. The MyD88-independent or TRIF-dependent pathway is activated exclusively by TLR3 and TLR4. Upon ligand binding, the TLR induces TRIF activation by interacting with TRAF6 and TRAF3. TRAF6 recruits a kinase that subsequently interacts with and activates the TAK1 complex, leading to the activation of NF-κB and MAPK and the induction of proinflammatory cytokines. On the other hand, TRAF3 recruits IKK-related kinases, which subsequently phosphorylate IRF3, promoting its translocation to the nucleus and inducing type I interferon gene expression. MAPK, mitogen-activated protein kinase; MyD88, myeloid differentiation primary response protein 88; NF-ĸB, nuclear factor ĸ light chain enhancer of activated B cells; TLR, toll-like receptor; TRIF, interferon-β-inducing TIR domain-containing adaptor protein. Image created with BioRender

TLR activation begins when an antigen binds to its corresponding TLR ligand, inducing either receptor dimerization or a conformational change in pre-existing dimers. This structural shift enables adaptor proteins to bind and trigger immune response. Among the adaptors recruited by TLRs, the myeloid differentiation primary response protein 88 (MyD88) and the TIR domain-containing adaptor protein inducing interferon-β (TRIF) are the most important ones (Fig. 3) [7]. The MyD88-dependent pathway is utilized by all TLRs except TLR3. Upon activation, the MyD88 protein binds to TIR domains of TLRs, facilitating their interaction with IRAK-4 and IRAK-1. This cascade activates MAP kinases and allows the nuclear translocation of the NF-κB dimer and the binding to response elements in target gene promoters. In contrast, the MyD88-independent signaling pathway is exclusive to TLR3 and TLR4 receptors, and relies on the adaptor protein TRIF. In this pathway, TRIF recruits a protein complex comprising TRAF6 and TAK1, which activates IKK kinases or MAPK. This process culminates in the release and nuclear translocation of NF-κB, initiating transcription of immune-related genes (Fig. 3). Furthermore, TRIF interacts with TBK1/IKK, triggering the translocation of nuclear factor IRF-3 to the nucleus and the synthesis of type I interferon [67].

The activation of TLRs through both pathways triggers numerous defense mechanisms including the release of reactive oxygen species (ROS), proinflammatory cytokines such as IL-6, IL-12, and TNFα, and peptides such as β-defensin 2 [12]. These responses drive immune cell recruitment of different types of immune cells, such as neutrophils, macrophages, and DC, the oxidative stress induction, and the establishment of an inflammatory microenvironment necessary for the pathogens elimination. Consequently, the elevated presence levels of proinflammatory cytokines such as TNFα, IL-6 or IL-1β can be taken as key biomarkers of TLR activation [67].

### Role of TLRs in food allergies

As previously discussed, the development of various allergies has been linked to environmental factors such as the type of delivery (cesarean or vaginal), maternal nutrition during pregnancy and lactation, urban or rural environment, stress, and toxic habits [66]. As an example, research into dietary pattern including components and characteristics of Mediterranean and other healthy diets (e.g., fresh fruit and vegetables, fish, olive oil, reduced meat intake and home-cooked foods) have been associated with reduced prevalence of allergic disease. This suggests that nutritional profile plays a more relevant role than the consumption of isolated nutrients [66].

However, current understanding of how these factors affect TLR’s role in FA is limited. Moreover, although the role of TLRs in sensitization and tolerance processes to ingested/contact allergens is well-established, the causal relationship between TLRs and FA, particularly regarding immune response modulation, remain poorly understood and require further investigation [55, 67].

Neonates and infants are significantly more susceptible to FA compared to adults. However, the extent to which innate immune responses to stimuli differ between neonates and adults, and how these differences contribute to the increased susceptibility in early life, has not yet been fully characterized [20, 60]. Several studies suggest that TLRs may play a key role in the age-dependent onset of FA, although conclusive evidence is still lacking. Kollmann et al. investigated how TLR-mediated innate immune responses differ between human neonates and adults. Their study found that neonates exhibit an enhanced capacity to promote Th17 and Th2-type responses against extracellular pathogens, while showing a diminished response to intracellular pathogens [30]. This differential mechanism has yet to be validated in the context of food antigens, which is essential for understanding age-related differences in susceptibility to FA. Additionally, emerging evidence suggests that TLR function undergoes postnatal maturation and that children developing allergic diseases may exhibit altered developmental patterns influenced by microbiota composition, genetic predisposition, and epigenetic influences during perinatal development.

### Food allergies, microbiota, and TLR

In recent decades, research into microorganisms inhabiting mucosal membranes has gained significant importance, revealing that the microbiota (microbiome) acts as a critical regulator of the three major integration systems: the nervous, the endocrine, and the homeostatic systems, with particular significance for the immune system modulation [24]. The intestinal microbiota, particularly the colonic microbiota, has gained particular significance. The existence of the microbiota-gut-pancreas-liver-brain axis is now well-established, carrying profound implications for human health [70].

Intestinal health depends on the abundance and proportionality of the different microorganisms that make up the intestinal biota. This microbial composition is influenced by several factors, including the mode of delivery (vaginal birth or cesarean section), the type of infant feeding (breast milk versus formula), area of residence (rural or urban), and both maternal and infant diets, among others [18]. The process of microbial colonization in the human intestine during childhood plays a crucial role in the maturation and epigenetic regulation of the immune system [24]. Factors affecting gut microbial colonization in early life have been shown to affect the development of atopic disorders, including FA.

A particularly interesting finding is that the overall composition of the gut microbiota (which includes both commensal and pathogenic bacteria) can modulate innate immune mechanisms, depending on the predominant structure of lipopolysaccharides (LPS), such as tetramers, pentamers, or hexamers of acylated lipid-A, present in the microbiota. Vatanen et al. [63] studied the intestinal microbiome development during the three first years of life in Northern Europe infants, and its possible relationship with the incidence of autoimmune diseases (e.g., Diabetes). They found that Bacteroides species were less abundant in Russians infants but predominate in Finn and Estonian infants. the dominant LPS in the microbiota of Finnish and Estonian infants was derived from Bacteroides, whereas in Russian infants, it originated mainly from *E. coli*. Notably, these two types of LPS are structurally distinct, with Bacteroides-derived LPS inhibiting innate immune signaling and promoting endotoxin tolerance [63].

Disruption of the original gut microbiota, known as dysbiosis, has been linked to the development of FA. In children with FA, dysbiosis may lead to immune imbalances between effector T cells and Tregs. Thus, microbial dysbiosis in germ-free mice has been shown to be associated with Th2 development and IgE production against food allergens. On the other hand, the high proportion of some microorganisms in the gut microbiota may lead the immune system towards a helper T cell (Th1) phenotype that protects against FA, rather than the Th2 cell phenotype that is linked to atopy during infancy and childhood [24, 68]. In this regard, it has been shown that dysbiosis at an early age can increase the risk of developing FA. For example, a low abundance of Bifidobacterium, Enterococcus, and Bacteroides species has been associated with greater susceptibility to FA. However, it has recently been shown that *Clostridium butyricum* can induce IL-10-producing macrophages in the intestine [21].

The critical role of gut microbiota composition in the development of FA is further highlighted by studies in which colonization of germ-free mice with the fecal microbiota from a healthy infant-rich in Bifidobacterium spp. and Bacteroides spp.-conferred protection against cow's milk allergy following sensitization with β-lactoglobulin [58]. These results were associated with reduced T cell reactivity towards allergen, increased FoxP3 + Treg, and reduced bacterial translocation into the lamina propria. Bifidobacterium breve potentially activated CD103 + intestinal DC to produce IL-10 and IL-27 in a TLR2-dependent manner to induce IL-10-producing Th1 cells, whereas colonization of germ-free mice with Bacteroides fragilis restored the Th1/Th2 balance and prevented intestinal inflammation by inducing IL-10-producing CD4 + T cells.

Given these findings, it is logical to further investigate the role of altered TLRs signaling in the development of allergic diseases. Current evidence supports the idea that genetic and environmental factors can lead to early variations in TLR function, and that this may affect adaptive immune development. TLRs play a critical role in antigen presentation and T cell activation, both of which are essential steps in allergic responses. Consequently, a reduction in overall microbial exposure and diminished TLR stimulation may lead to weak Th1 imprinting and exaggerated Th2 responses, potentially increasing the risk of FA [71].

Thus, further studies are needed to assess the normal ontogeny of TLR function and possible differences in atopic infants. It is plausible that reduced TLR stimulation during infancy could contribute to the pathogenesis of allergic diseases by promoting uncontrolled Th2 responses. Pioneering studies in germ-free mice suggested that intestinal bacteria or LPS-like products were important for the generation of tolerance to dietary antigens [24]. Thus, Kiyono et al. [29] pointed out that oral tolerance might not occur in LPS-hyporesponsive mice such as C3H/HeJ. Later, Li et al. [36] defined the C3H/HeJ mouse as a model for systemic anaphylaxis to peanuts, and that this FA was associated with the lack of activation of the TLR4 signaling pathway [5, 34]. The same authors subsequently hypothesized that the ligand for TLR4 was produced by commensal bacteria and that a reduction in the bacterial load in newborn mice by using broad-spectrum antibiotics induced an allergic response equivalent to that observed in the TLR4 mutant mouse [5]. Similarly, Prescott et al. [56] compared TLR function in allergic and non-allergic infants after their first year of life (diagnosed with food allergy or atopic dermatitis) and found that the non-allergic group exhibited lower TLR-mediated responses than the allergic group, particularly in response to TLR3 ligands (lower concentrations of TNFα and IL-12) and TLR5 ligands (lower concentrations of TNFα and IL-10), with similar trends observed for other ligands. These findings suggest that lower TLR responses may be protective, consistent with observations that the beneficial effects of microbial exposure are associated with reduced TLR-mediated immune responses. [14, 24]. However, the significance and underlying mechanisms of these relationships remain to be fully elucidated. Thus, while alterations in TLR function appear to be associated with the development of allergic disease, the initial hypothesis (that reduced TLR function predisposes to allergic disease) appears overly simplistic.

Several publications suggest the importance of early stimulation through TLRs to regulate the possible development of FA. For example, consumption of human milk, as opposed to formula, has been associated with differential TLR expression (with downregulation of TLR2 and TLR3 function and upregulation of TLR4 and TLR5) This suggests that human milk, with its unique composition of proteins, fatty acids, glycerides, and prebiotic and probiotic compounds, distinctly shapes the infant gut microbiota, thereby influencing TLR ligand presentation and immune development [22, 34]. Thus, although we have not found specific evidence, in terms of early stimulation of the TLR system the importance of the consumption of a plural diet rich in plant-based foods and fiber, and bioactive compounds (e.g. Mediterranean diet) by the pregnant mother can be suggested as it has been found to positively affect several negative pregnancy outcomes and some mothers'markers of degenerative diseases (e.g. inflammation, dyslipemia, insulin resistance, immune function, metabolic syndrome) due to the microbiota eubiosis generated [31, 41]. It is plausible that the maternal microbiota, or its metabolites, are transferred to the fetus, contributing to fetal health and optimal TLR function. In fact, two different pathways have been proposed by which the maternal gut microbiota may influence the fetus: a) direct colonization of fetal tissues (e.g. gut) by maternal intestinal microbes; b) transplacental transport of microbiota-derived metabolites (e.g. SCFAs) and/or transplacental transport of microbiota-derived microbial compounds (such as LPS, flagellin) [44].

Recent studies also indicate that the immunomodulatory properties of omega-3 polyunsaturated fatty acids (particularly eicosapentaenoic acid, C20:5 n-3; abundant in fish oils) are mediated, at least in part, by TLR4 [47]. Although clear evidence of FA reduction by omega-3 fatty acid consumption is not available yet, it has been suggested that eicosapentaenoic and docosahexaenoic acid supplementation during pregnancy could modulate the newborn’s immune system before allergic responses to food antigens are established, especially in infants genetically predisposed to IgE-mediated allergies. Animal studies further support that omega-3 fatty acid intake can beneficially alter gut microbiota-derived metabolites associated with tolerance mechanisms, thereby decreasing allergic sensitization [19, 32].

The importance of late stimulation through TLRs to regulate the possible development of FA is also little known. In adult experimental animals, an association between the composition of the microbiota and the expression of TLRs has also been shown. In this regard, it is known that in FA, the relationship between the microbiota and the host is carried out through the TLR-PRR recognition system, and that TLR4 and TLR9 are the ligands that have shown a greater association [5].

TLRs are pivotal in recognizing components of the microbiota and play a key role in maintaining immune tolerance or, conversely, inducing immune responses. They serve as the primary communication channel between the host and its microbiota, helping to sustain tolerance toward commensal organisms. TLRs are able to discriminate between healthy colonization and the presence of pathogens. In this sense, it has been suggested that an efficient crosstalk between microbiota and TLRs induces low responsiveness state against the commensal microbiota, through different strategies to regulate the interaction between bacterial signals (resident and pathogens) and TLR, allowing intestinal discrimination between these two types of microbes to keep the tolerance toward the local microbiota, preventing from inflammation [33]. Le Noci et al. describe five discriminative strategies that allow the host to differentiate between commensal and pathogenic bacteria: tolerance by exclusion (commensals help tighten epithelial junctions via TLR activation, enhancing barrier integrity), specific compartmentalization of TLRs (certain TLRs and co-receptors are compartmentalized to recognize bacterial signals only if they penetrate the epithelium), TLR-commensal signaling shaping immune responses (this signaling influences immune responses through intra-epithelial T cells producing protective factors when mucosal integrity is compromised), varying affinities of PAMPs for TLRs (variations in PAMPs enable TLRs to preferentially recognize pathogenic signals), and the requirement of a second signal for immune activation (based on the propose that immune responses require two signals: first provided by TLR agonists from both commensals and pathogens, triggering NF-κB-mediated pro-IL-1β production; and second, a danger signal exclusively from virulent pathogens causing cell stress, enabling the immune system to differentiate between harmless commensals and potentially dangerous pathogens) [33].

Moreover, both the composition of the microbiota and the expression of TLRs are regulated by the circadian clock [65]. *Vici versa*, the microbiome stabilizes circadian rhythms in the gut [72]. Circadian regulation affects the severity of the inflammatory response and the interaction between the microbiota and TLRs in the gut. Additionally, various immune functions-including cytokine production and the regulation of intestinal permeability-also follow a circadian pattern. Disruption of these rhythms, known as chronodisruption, has been shown to increase microbial translocation and compromise epithelial tight junctions. In summary, the circadian clock plays a crucial role in regulating the immune response and intestinal homeostasis. Beyond TLR expression, cytokine production by macrophages and CD4 + T cells are also under circadian control [9] as are the suppressor function of FoxP3 + Treg cells, leukocyte trafficking, and antibody production [27]. It is therefore possible that rising incidence of FA observed in Western societies will be partially attributed to the increasing prevalence of chronodisruption, which is well recognized as a contributor to low-grade inflammation in many common chronic diseases.

#### Genetic and epigenetics, food allergies, and TLRs

a) Genetic effects: The physiometabolic response to diet [13], foods, and bioactive compounds [10] varies among individuals according to their genetic and epigenetic makeup. Although current evidence on genetic factors influencing FA is still limited, some studies suggest that functional variations in aspects of microbial TLR signaling are associated with clinical phenotypes and disease susceptibility. To date, there is more information on the relationship of genetic variables than epigenetic variables with FA. For instance, mutations in genes located in the major histocompatibility complex (MHC) have been found to exert very important effects, while mutations in genes that encode TLRs, as well as genes that encode the TLR activation cascade (e.g. CD14), and production of pro- or anti-inflammatory cytokines (interleukins, NF-κB, etc.), or the integrity of the digestive barrier (occludin, zonulin, etc.) appear to be less influential or have not yet been thoroughly studied [15]. In fact, in the magnificent review by Kanchan et al. [26] where a broad study of overlap between FA, asthma, allergic dermatitis and allergic rhinitis was carried out, the information on TLR gene polymorphisms was found to be scarce, with associations primarily limited to TLR1. Despite considerable heterogeneity in study designs, definitions of FA, and relatively small sample sizes (n < 200), there is clear evidence that certain loci, genes, and polymorphisms are at least associated with FA risk. The implicated genetic loci broadly suggest roles for genes involved in intestinal barrier integrity and immune function [22].

Regarding *TLR* variants, different authors have found a reduced response to bacterial LPS in carriers of different *TLR4* polymorphisms, as well as a lower induction of the cytokines IL-12 and IL-10 after stimulation with LPS. Additionally, maternal allergy risk has been linked to reduced neonatal TLR2 responses and lower levels of TLR2, TLR4, and CD14 mRNA in cord blood, leading to the hypothesis that hereditary predisposition to TLR immaturity may contribute to less effective inhibition of TLR-mediated allergic responses. However, these same authors found opposite results in a later study [1, 16, 55].

Although polymorphisms in *TLR2* and *TLR4* have been associated with changes in TLR and, consequently, with allergy risk, a direct causal relationship between these genetic variants and FA has not yet been established, possibly due to small sample sizes. Thus, Galli et al. [17] performed candidate gene association studies with negative results for *TLR* (*TLR2* and *TLR4*). Nonetheless, the exclusion of the role of TLRs in FA still seems inadvisable. By the way, using coupling of candidate gene association studies and chromosomal microarrays that detect copy number variants, an association for different FA and *TLR10/1/6* and *IL-2* has been identified [22]. Further details on the functions of these candidate genes in relation to FA are provided in the study by Kanchan et al. [26]. Poole et al. [54] observed that children of farmers exposed to a greater number of pathogens during their developmental stages, had a lower risk of developing FA by presenting a higher expression of TLR2 mRNA. Poole et al. observed that children with nut allergy have lower expression of TLR2 mRNA and mRNA TLR4.

Beyond TLR polymorphisms, some studies have reported associations between FA and polymorphisms related to intestinal barrier integrity and the innate immune response. For example, polymorphisms in the gene encoding *CD14* have been linked to total serum IgE levels, suggesting a functional relationship between atopy and TLR4 pathways. However, discrepancies regarding *TLR4* genetic polymorphisms and their beneficial or detrimental role in allergies have been reported [1, 5, 16, 54, 56]. CD14 plays an important role in the innate immune system as part of the protein complex that binds bacterial LPS. Notably, the *CD14* SNP rs2569190 was associated with peanut allergy in a study of 53 peanut-allergic children and their peanut-tolerant siblings [25].

b) Epigenetic effects: it is known that different processes (such as DNA methylation, histone acetylation/methylation, presence of microRNAs) can affect the transcriptome and proteomes by modifying gene expression and protein formation, without implying modification of the DNA nucleotide sequence [38]. The presence of food antigens is critical for maintaining a high population of Tregs in the intestine. Hypomethylation of the FoxP3 locus in Tregs has been proposed as a key epigenetic mechanism enhancing transcription during oral tolerance development [71]. Poole et al. [54] also found a significant correlation between TLR2 methylation and TLR2/CD14 expression in children with nut allergy, in whom the resulting change in gene expression was greater. In relation to TLR9, unmethylated versus methylated CpG signaling through this receptor could help differentiate a healthy microbiota from invading bacteria and promote the generation of tolerogenic DC and Tregs [37]. In addition, Lorite et al. [38] suggested that pregnancy dietary fiber could promote beneficial effects on maternal microbiota, which could be transferred to the offspring, improving some metabolic syndrome markers through methylation of TLR2 and TLR4 genes that in turn would affect the maturation of immune systems and the inflammatory cytokine production. These findings indicate that methylation of the promoter region plays a role in the general regulation of the expression of these genes and that it could be a potential therapeutic target for FA.

On the other hand, it was found that TLR4-related signals induced by gut microbiota inhibit the development of reactions against food antigens [5]. While several single nucleotide polymorphisms (SNPs) in TLR4 (e.g., rs4986790, rs4986791) and TLR2 (e.g., rs1898830, rs5743708) have been identified, their potential association with food allergy remains understudied [43, 67].

### Treatment of food allergy through TLRs

Currently, there is no curative or disease-modifying treatment for FA approved by international regulatory agencies. As a result, any approach that can block uncontrolled immune responses-particularly those involving the activation pathways of TLRs, especially TLR2 and TLR4-appears promising. The new advances in TLR-signaling pathways and their possible involvement in FA are allowing the development of targeted therapies that modulate these pathways. Zhu et al. [73] demonstrated that oral administration of a synthetic TLR9 agonist (an immunomodulatory oligonucleotide) can promote a strong Th1-type immune response and reduce Th2-response by stimulating TLR9 at both mucosal and systemic levels, thereby protecting against anaphylactic reactions in murine models of peanut allergy.

Another strategy evaluated to modulate the allergen-specific immunomodulatory is the covalent conjugation of allergens with TLR agonists. Thus, Losada-Mendez et al. [39] synthesized ligands conjugated with a T cell peptide from the peach allergen and administered them to patients allergic to this fruit. These authors demonstrated that said conjugate was captured by DC and presented to immune cells, promoting the induction of Treg cells and reducing the effector response. Finally, the administration of probiotics promotes an adequate reaction of DC that allows the differentiation of Th0 cells to Treg, which exerts an inhibitory effect on the inflammatory response of Th1, Th2 and Th17 by activating TLR2 in DC [53].

Lastly, emerging evidence highlights a complex and dynamic interaction between TLR signaling and the immunological changes induced by OIT for FA. As previously mentioned, the induction of immune tolerance during OIT involves multiple mechanisms that suppress both early and late-phase inflammatory responses following exposure to food allergens [28]. In addition to antibody-mediated effects, a key marker of successful tolerance induction is the reduction in allergen-specific circulating Th2 cells and decreased local production of Th2-associated cytokines such as IL-4, IL-5, and IL-13 upon allergen challenge. OIT has also been shown to modulate TLR expression on DC, leading to diminished secretion of proinflammatory cytokines and promoting the differentiation of Tregs, while concurrently inhibiting the expansion of Th2 cells, central mediators of allergic responses [40]. Clinical trials investigating peanut OIT have demonstrated that treatment can modulate TLR-dependent pathways in DC, decreasing inflammatory cytokine output and enhancing regulatory immune responses [2].

Collectively, these findings suggest that a deeper understanding of TLRs may lead to the development of novel immunotherapies, such as vaccines designed to reprogram the immune system to tolerate food allergens. These vaccines could use TLR agonists or antagonists to induce protective rather than allergic immune responses. To date, TLR agonists have been developed to treat allergies by upregulating the innate immune system, while TLR antagonists have been used to manage various inflammatory and autoimmune conditions. However, evidence supporting their use in FA remains limited.

## Conclusions and future remarks

Despite significant advances in immunonutrition, our understanding remains incomplete regarding why, despite widespread exposure to food allergens, only a few people experience adverse immunological reactions, while the majority exhibit varying degrees of immunological tolerance. Another area that is not fully understood is the marked increase in the prevalence of various allergies-including food allergies-over recent decades, which manifest primarily as cutaneous, respiratory, and digestive symptoms. Among many aspects, the hypothesis of hygiene, type of delivery, breastfeeding, contamination, and early introduction of foods that are foreign to our gastronomic, and cultural heritage have been put forward. Currently, the primary treatment for food allergies is the elimination of the offending food from the diet. Other therapeutic approaches, such as vaccines, desensitization protocols, and immunological blockers, have also been explored. However, the precise mechanisms underlying the development of sensitization or tolerance to specific foods or food components remain unclear, as does the reason why food allergies can sometimes emerge in adulthood. This highlights the need to further unravel the complex immunological processes involved. Advances in our understanding of the microbiota, the various types of Toll-like receptors (TLRs), and their relationship with Th1 and Th2 immune responses in food allergy have been significant. Nevertheless, it remains unclear how the abundance and variety of the microbiota affects the “metabolization” of microbial membrane LPS in the environment or present in food, how they would affect the start of sensitization and which microorganisms or the interactions between them best condition immunotolerance or immunosensitization.

Equally important are the differences in allergen responses observed among individuals exposed to similar environmental conditions. This variability is influenced by nutritional genomics, including nutrigenetics (differences in TLR responses and signaling pathways due to gene polymorphisms) and epigenetics (modifications affecting gene transcription and translation). Therefore, the increasing application of genome studies and ‘omic’ sciences is urgently needed to have a broad and specific framework of the metabolic implications of TLRs and their application in both precision medicine and nutrition for the treatment and prevention of FA. One of the most significant advances that may contribute to the study of TLRs is exfoliome analysis. This represents a revolutionary approach in non-invasive diagnostics, leveraging naturally shed epithelial cells to analyze the transcriptome, which could have important implications for advancing research, diagnosis, and monitoring of TLR-related changes [57]. Additionally, the role of gender differences in the prevalence and types of FA are also not well known. Information on the benefits of not including allergens during pregnancy in preventing future FA has also been widely discussed. Therefore, we believe that future studies will be addressed to:

- Determine the minimum concentration and number of exposures required to induce sensitization to food allergens, as well as the conditions that favor this process.
- Delve deeper into the convenience of early or late introduction of foods other than breast milk in relation to sensitization.
- Elucidate the mechanisms that lead to desensitization or cross-tolerance, particularly those involving the suppression of pro-inflammatory or anti-inflammatory cytokine release.
- Characterize the most common microbiome profiles (including fungiome or mycobiome and virome,) in the different FA dependent or independent of IgE and clarify the role of different bacterial phyla in these conditions.
- Define the role of the different types of TLRs in the most prevalent FA, with respect to both plant-based and animal-based foods.
- Decipher the role of different types of TLR receptors in phagocytic and DC modulating the secretion of immunoregulatory factors relevant to FA
- Further explore the bidirectional relationship between the microbiota and TLRs in the context of different types of FA.
- Study the possible relationship between pathologies that present TLR modulation, such as obesity, lupus or asthma, and the increased presence of FA adults.
- Clarify the role of probiotics in modulating the microbiota-TLR axis and their potential benefits in promoting tolerance and desensitization to food allergens.
- Identify candidate genes and epigenes, as well as their most common polymorphisms, involved in FA in children, women, and men
- Initiate through extensive genome studies the application of weighted and unweighted Genetic Risk Scores (GRP) for various FA across different populations, including infants, children, women, and men.

Given the potentially negative implications that the withdrawal of a food or ingredients in those where the allergen is found may have, look for nutritional palliative measures to compensate for the marginal or severe malnutrition that may occur. Additionally, there is an urgent need to better understand how the development of vaccines and novel treatments may influence TLRs activity, their interactions with the microbiota, and the differences between allergic and non-allergic individuals based on their genome, epigenome, and metagenome, while also taking into account gonadal and chromosomal sex.

Therefore, elucidating the precise mechanisms underlying these conditions, identifying specific biomarkers for early diagnosis, and expanding our knowledge of their relationship with TLRs could lead to more effective interventions. Such advances have the potential to reduce disease burden and improve the quality of life for affected individuals. This knowledge can influence health policies, promoting evidence-based approaches to the management and prevention of FA, from dietary recommendations to food allergen regulations. Ultimately, these insights open the door to more targeted and effective treatments that can minimize allergic reactions without suppressing the entire immune system, thereby reducing potential side effects.

## Data Availability

No datasets were generated or analysed during the current study.
